# Measurement invariance of the comprehensive feeding practices questionnaire in dual-earner parents with adolescent children in Chile

**DOI:** 10.3389/fpsyg.2022.1031391

**Published:** 2023-01-04

**Authors:** Carola Del Valle, Horacio Miranda, Ligia Orellana, Klaus G. Grunert, Berta Schnettler

**Affiliations:** ^1^Doctorado en Ciencias Agroalimentarias y Medioambiente, Facultad de Ciencias Agropecuarias y Medioambiente, Universidad de La Frontera, Temuco, Chile; ^2^Departamento de Producción Agropecuaria, Facultad de Ciencias Agropecuarias y Medioambiente, Universidad de La Frontera, Temuco, Chile; ^3^Universidad de La Frontera, Centro de Excelencia en Psicología Economía y del Consumo, Núcleo Científico Tecnológico en Ciencias Sociales, Temuco, Chile; ^4^Departamento de Psicología, Facultad de Educación, Ciencias Sociales y Humanidades, Universidad de La Frontera, Temuco, Chile; ^5^Department of Management, Aarhus University, MAPP Centre. Aarhus, Denmark, and University of Vaasa, Vaasa, Finland, Denmark; ^6^Universidad de La Frontera, Scientific and Technological Bioresource Nucleus (BIOREN-UFRO), Temuco, Chile; ^7^Facultad de Especialidades Empresariales, Universidad Católica de Santiago de Guayaquil, Guayaquil, Ecuador

**Keywords:** food parenting practices, CFPQ, eating habits, psychometric properties, multigroup measure invariance

## Abstract

The Comprehensive Feeding Practices Questionnaire (CFPQ) has been evaluated in international studies, but the evaluation of its psychometric properties in Spanish, and in particular for parents of adolescents is still limited, and the invariance of measurement according to gender has not been evaluated. Therefore, the objectives of this study were: (1) To adapt the five-factor model of the CFPQ instrument to Spanish; (2) To examine the psychometric properties of this adaptation; and (3) To evaluate the measurement invariance of the model to verify the equivalence of measurement of the perceptions of food parenting practices between mothers and fathers belonging to nuclear, dual-earner families with adolescent children. Participants were 946 mothers and fathers from Southern Chile. Results showed that the conceptual equivalence for the CFPQ was achieved. An exploratory factor analysis was performed for a five-factor model: Monitoring, Child control, Restriction for weight control, Modeling and Environment. Horn’s parallel analysis identified four factors, while factor loading analysis determined the removal of the environment factor. Confirmatory factor analysis presented good reliability values. Convergent and discriminant validity was confirmed, and medium-to-high levels of goodness of fit were obtained, eliminating two items. Results supported a final model of four factors and 19 items. Multigroup analysis of the measurement model verified the configural and metric invariance between fathers and mothers, while the scalar and strict partial invariance was verified. These findings are a relevant guide to measure factorial scores in the four-factor model of the CFPQ, establishing a characterization of feeding practices of parents and adolescents.

## 1. Introduction

Parents are the main responsible for feeding their family (Piccoli et al., 2017), and thus they adopt behavioral strategies to maintain, modify or control their children’s eating habits. These behavioral styles are known as Food Parenting Practices (FPP; Rodríguez Arauz and Ramírez, 2017), which are strongly related to the eating habits of parents and the type of food they offer to their children (Kaar et al., 2016). Research has shown that FPP influence eating patterns and body weight of children (Haszard et al., 2013; González-Torres et al., 2018; Saltzman et al., 2018). Some FPP may have negative consequences even in future generations (e.g., on weight, obesity and associated chronic non-communicable diseases), because the eating habits acquired in childhood and adolescence can be perpetuated and replicated when these children become parents (Nowicka et al., 2014; Corsini et al., 2018). The inappropriate use of FPP is, in part, because parents do not have sufficient knowledge and are unaware of the changes they must consider in the different stages of their children’s lives (Castrillón and Giraldo Roldán, 2014; González-Torres et al., 2018; Russell et al., 2018).

To evaluate FPP, instruments are needed that measure the different FPP that fathers and mothers apply to their children. Vaughn et al. (2016) distinguished three constructs in FPP: coercive control, structure, and support for autonomy. Coercive control refers to restriction, pressure to eat, threats and bribes, and the use of food to control negative emotions. Structure refers to factors such as modeling, monitoring, rules and limits, meal and snack routines, food availability and accessibility, and food preparation. Lastly, support for autonomy is about nutritional education, children’s participation, encouragement, praise, reasoning, and negotiation. For measuring FPP, the Comprehensive Feeding Practices Questionnaire (CFPQ) is currently one of the most widely used instruments. The CFPQ (Musher-Eizenman and Holub, 2007) was designed to measure FPP directed at children, and it is made up of 12 factors and 49 items; it has subsequently been adapted for use with parents of adolescents Melbye et al. (2011). Ángel et al. (2021) translated this questionnaire into Spanish to be answered by Mexican mothers with preschool-aged children.

Researchers have aimed to validate the original factorial structure of the CFPQ in parents of preschoolers, schoolchildren, and adolescents. Results have been unstable, however, and the original structure has not been replicated, obtaining instead fewer factors in the available studies (Melbye et al., 2011; Haszard et al., 2013; Mais et al., 2015; Al-Qerem et al., 2017; Piccoli et al., 2017; Saltzman et al., 2018; Arlinghaus et al., 2019). Reducing the number of dimensions of the CFPQ has improved the consistency across studies applying it. In New Zealand, in a sample of 1,013 parents of children aged five to 8 years, Haszard et al. (2013) found a good fit for the CFPQ with a five-factor model (i.e., Monitoring, Pressure, Restriction for weight control, Child control and Healthy eating guidance). In Brazil, Mais et al. (2015) worked with a sample of parents of school-age children, and identified a six-factor model (i.e., Healthy eating guidance, Monitoring, Restriction for weight control, Restriction for Health, Emotion regulation and Pressure). In Jordan, in a sample of mothers with children aged 6–12, Al-Qerem et al. (2017) found a 11-factor model (i.e., Monitoring, Child control, Emotion regulation, Environment, Involvement, Pressure, Restriction for weight control, Food as reward, Restriction for Health, Modeling and Teach and encourage). In the United States, with a sample of 260 mothers with children of preschool age, Saltzman et al. (2018) conducted a longitudinal study using a seven-factor model at time one (T1): Monitoring, Restriction for weight control, Pressure, Involvement in nutrition, Emotion regulation, Food as reward and Healthy eating guidance, and at time two (T2) a model of five factors: Monitoring, Pressure, Restriction for health, Restriction for weight control and Healthy eating guidance. With a sample of 187 Hispanic American mothers residing in the United States with children between the ages of three and five (79% of the questionnaires were completed in Spanish), Arlinghaus et al. (2019) arrived at factorial structure of the CFPQ with a significant reduction in the number of items and factors. These authors validated a five-factor model with 34 items: Monitoring, Restriction for weight control, Promotion of overconsumption (this factor covered the following factors from the original CFPQ: Emotion regulation, Child control, Food as a reward and Pressure), Healthy eating guidance (Haszard et al., 2013; Mais et al., 2015; Warkentin et al., 2016; Saltzman et al., 2018; Arlinghaus et al., 2019) and finally Healthy food variety. Finally, the adaptation of the CFPQ by Melbye et al. (2011) found 10 factors with 42 items.

These results have led researchers to emphasize the need for further research on the factorial structure of the CFPQ in different countries and contexts (Haszard et al., 2013; Saltzman et al., 2018; Arlinghaus et al., 2019), to determine whether the factorial structure of this instrument is maintained or changed according to cultural differences and the age of the children (Melbye et al., 2011, 2012; Doaei et al., 2013; Haszard et al., 2013; Mais et al., 2015; Rodríguez Arauz and Ramírez, 2017; Saltzman et al., 2018; Arlinghaus et al., 2019). Most of the research has focused on preschool and school children, because at this stage parents have a strong influence on the eating habits of their children (Rodríguez Arauz and Ramírez, 2017; Monnery-Patris et al., 2019). However, parents also face significant challenges in developing or maintaining healthy eating habits when their children become adolescents.

Adolescence is marked by high emotional instability (Benarroch et al., 2017; Piccoli et al., 2017), and adolescents acquire greater autonomy in various aspects of life including their diet, which can render the FPP used by their parents less effective (Schnettler et al., 2018c). Therefore, during this stage, the influence of parents on their children’s nutrition decreases and the influence of the media and peers increases (Melbye et al., 2011) which in part explains the decrease in diet quality and increased risk of weight gain during adolescence (Gunther et al., 2019; Balantekin et al., 2020). These effects, in turn, can lead to negative consequences for the physical and psychosocial health and quality of life of adolescents (Piccoli et al., 2017; Lopez et al., 2018). Hence, adolescents still need their parents to model and guide their eating habits (Piccoli et al., 2017).

To the best of our knowledge, the CFPQ version for parents of adolescents Melbye et al. (2011) has not been adapted to other languages, and its psychometric properties have not been evaluated in Spanish-speaking countries. Therefore, the first aim of this study is to adapt the Melbye et al. (2011) version of the CFPQ to Spanish, accounting for the factors that have shown greater stability in previous studies (Haszard et al., 2013; Mais et al., 2015; Saltzman et al., 2018; Arlinghaus et al., 2019), that is, Monitoring, Child control, Restriction for weight control, Modeling and Environment. The choice of these factors is also based on research that accounts for the use of these FPP by parents of adolescents: Monitoring (Gunther et al., 2019; Costarelli et al., 2021), Child control (Schnettler et al., 2018b,c), Restriction for weight control (Loth et al., 2016; Haycraft, 2020; Lister et al., 2020), Modeling (Loth et al., 2016; Garrido-Fernández et al., 2020; Jaeger et al., 2021) and Environment (Loth et al., 2016; Gunther et al., 2019).

Research on how fathers and mothers exercise co-parenting food practices is scarce (Tan et al., 2016). Studies suggest that mothers continue to be the main responsible for raising and feeding their children (Garrido-Fernández et al., 2020; Zhang et al., 2020; De-Jongh González et al., 2021; Schnettler et al., 2021). However, other research indicates that the involvement of fathers in feeding their children has increased, due in part to the incorporation of mothers into the workforce (Cho and Coulton, 2016; Vaughn et al., 2016; Cladellas Pros et al., 2017; Sharif et al., 2017; Rahill et al., 2020; Schnettler et al., 2021). Furthermore, in families with dual-earner parents, limited time availability and conflicting work schedules have been found to be the main obstacles to promoting healthy eating behaviors in adolescents (Schnettler et al., 2018d; Liu and Grunert, 2020). Dual-earner parents thus face specific challenges when exerting FPP to their adolescent children, while adolescents are attuned to their parents’ work, and can show concern and participate in work-related experiences of their parents (Orellana et al., 2021). Therefore, this study focuses on the adaptation of the five-factor CFPQ in dual-earner parents of adolescents.

Lastly, no available studies have evaluated the measurement invariance according to the parents’ gender. This is a relevant issue because evidence indicates that fathers and mothers have a different role in the formation of their children’s eating habits (Frankel and Kuno, 2019). Although the available literature indicates that there are similarities in how fathers and mothers exert FPP (Rahill et al., 2020; De-Jongh González et al., 2021), it has also been shown that, compared to mothers, fathers’ influence can be more decisive in the eating habits of their children (De-Jongh González et al., 2021), and that fathers apply FPP more coercively (Rahill et al., 2020). Therefore, it is possible to hypothesize that mothers and fathers have a different understanding of FPP and of their possible effects on their children.

Against this background, the objectives of this study were: (1) To adapt a five-factor model of the CFPQ (Melbye et al., 2011) to Spanish; (2) To examine its psychometric properties; and (3) To evaluate the measurement invariance of the model to verify the equivalence of measurement of FPP between mothers and fathers in dual-earner families with adolescent children.

## 2. Materials and methods

### 2.1. Sample and procedure

The population was defined as nuclear families with two parental incomes and adolescent children between 12 and 16 years old. Participants were recruited according to a non-probability sampling design by quotas which are proportional to the communal distribution of families according to the Socioeconomic Level (high, medium, and low) in the city of Temuco to obtain a sample that reflects a varied socioeconomic level. 946 participants corresponding to 473 mothers and 473 fathers from two-parent families were recruited in 2019. This study is part of a wider research project on the relations between work, family, and food-related life in Chilean families (Schnettler et al., 2018a). Sample size was determined considering 10 participants for each item of each scale used in this research project. This criterion is based on statistical simulation research developed by Gagne and Hancock (2006) who proposed 7–12 participants per item, in accordance with Kyriazos (2018), who proposed working with ten participants per item.

Participants were recruited in seven schools located in the urban area of the city, and which served socioeconomically diverse populations. Principals from each school signed authorization letters to conduct the research among their students and to support recruiting efforts between the research team and the students and their families. Participants were contacted *via* the schools with an invitation to participate in this study, and received the following information: Aims of the study, sample criteria, the questionnaire structure and data collection procedure, and the anonymous and confidential treatment of the data. Prior to data collection, the parents were asked to sign an informed consent form to confirm their participation and their understanding of the confidentiality and anonymity of their responses. Those families in which both parents agreed to participate were visited in their homes by trained interviewers (Psychology undergraduate students in the final years of the program). After the parents signed the informed consent form, the interviewers personally administered the questionnaires separately to the mother and father; interviewers were trained avoid introducing bias in the participants’ responses. The interviewers recorded participants’ responses in the online survey platform QuestionPro (QuestionPro Inc) using tablets to reduce the risk of data transcription errors. After answering both questionnaires, each couple received a gift card worth approximately USD 15. The Ethics Committee of the University of La Frontera approved the study protocol (Protocol Number 007/2019).

### 2.2. Instrument

The Comprehensive Feeding Practices Questionnaire, Musher-Eizenman and Holub (2007) was originally designed to measure infant FPP, and it is composed of 49 items grouped into 12 factors. In the present research, we use the adaptation by Melbye et al. (2011) for parents of adolescents. Measured 10 factors with 42 items. The participants responded to five factors: Monitoring, Child control, Restriction for weight control, Modeling and Environment of the adaptation. These authors obtained the following Cronbach’s alphas for each factor studied; Monitoring = 0.84, Child control = 0.55, Restriction for weight control = 0.83, Modeling = 0.66 and Environment = 0.57. According to Melbye et al. (2011), the Monitoring factor assesses when a parent tracks the unhealthy foods their child eats. The Child control factor assesses how much parents allow the child to make decisions about what and when to eat. The restriction for weight control factor assesses how much a parent restricts or controls their child’s eating. The Modeling factor assesses parents actively demonstrate healthy eating for the child and the Environment factor assesses how parents make (un)healthy foods available in the home (Supplementary material).

The items of the Child control and Monitoring factors were answered with a 5-point Likert-type scale, from 1: “never” to 5: “always.” The remaining factors (Restriction for weight control, Modeling and Environment) were answered with a 5-point Likert-type scale, from 1: “totally disagree” to 5 “totally agree.”

A group of experts translated the items into Spanish and evaluated their conceptual equivalence. All items from factors Restriction for weight control, Modeling and Environment (including Mod47) were changed from first person in English to third person in Spanish to be consistent with the items from the Monitoring and Child control factors. The instrument was then piloted with 131 participants representative in terms of gender and age of the target population. The clarity of the items was confirmed. The questionnaire also included questions for the sociodemographic classification of the family (Table 1).

**Table 1:** Sample characteristics: centralization, dispersion and value of *p*.

Variable analyzed (*n* = 946)	Value	Value of *p*
Age [Mean (SD) (Range)]:		
Mother	39.1 (7.2) (22–60)	–
Father	42.0 (8.9) (20–95)	0.001^1^
Socioeconomic status (%):		
High	22.2	–
Middle	61.5	–
Low	16.3	–
Gender of the main breadwinner (%):		
Female	23.3	–
Male	76.7	0.001^2^
Gender children’s (%):		
Female	48.6	–
Male	51.4	0.232^2^
Mother’s educational level (%):		
Primary school incomplete	0.8	–
Primary school complete	2.1	–
High school incomplete	6.8	–
High school complete	36.8	–
University incomplete	27.3	–
University complete	21.8	–
Post graduate studies	4.4	–
Father’s educational level (%):		
Primary school incomplete	1.1	–
Primary school complete	5.1	–
High school incomplete	13.1	–
High school complete	41.2	–
University incomplete	18.6	–
University complete	16.7	–
Post graduate studies	4.2	–
Number of family members [Mean (SD)]	4.4 (1.0)	–
Number of children [Mean (SD)]	2.2 (0.8)	–
Children’s age [Mean (SD)]	12.5 (1.7)	–

^1^Independent simple *t*-test. Two value of *p* corresponds to the (bilateral).

^2^Value of *p* corresponds to the asymptotic significance obtained in Pearson’s Chi-square test.

### 2.3. Data analysis

For the descriptive analysis, the Statistical Package for Social Sciences (IBM SPSS) v. 23 was used. For the exploratory factor analysis (EFA; Lloret-Segura et al., 2014), confirmatory factor analysis (CFA; Marsh et al., 1998) and the multigroup factorial invariance analysis of the measurement model between samples (Svetina et al., 2020), the software Mplus v. 7.4.

The purpose of the EFA was to identify the items and the number of latent factors to retain in the five analyzed factors of the instrument, to carry out a validation using the two-sample system. For this purpose, a subsample of 284 participants was drawn, corresponding to 30% of the total sample. Before performing the EFA, it was verified whether the empirical correlation matrix had the characteristics required for this analysis. The Kaiser-Meyer-Olkin (KMO) index (≥ 0.7) was used to evaluate the sampling adequacy. To evaluate the appropriate level of correlations, the Bartlett sphericity hypothesis test (≤ 0.05) was applied (Williams et al., 2010) and to detect the presence of multicollinearity, the determinant of the correlation matrix (> 0.0). To estimate the saturations, the EFA was considered an ordinal response scale of the items (Rhemtulla et al., 2012), due to this the polychoric correlation matrix (Olsson, 1979) and the weighted robust least squares estimation method (WLSMV; Li, 2016) were used. Items with loadings < 0.30 were eliminated. The number of factors to retain in the model was determined by applying Horn’s parallel analysis (Horn, 1965), using the 25 items of the five factors initially proposed from the CFPQ.

Once the number of factors had been identified and the items to be retained were selected in the EFA stage, a CFA was performed on a subsample of 662 participants corresponding to the remaining 70% of the total sample. Items with factor loadings < 0.40 were eliminated (Pituch and Stevens, 2016). The psychometric reliability index was then estimated using McDonald’s Omega coefficient (McDonald, 2013). The convergent validity was determined by the statistical significance of the saturations (*p* ≤ 0.05; Marsh et al., 2013). The discriminant validity between two latent factors was verified when the squared correlation was lower than the mean extracted variances (AVE) of the factors (Fornell and Larcker, 1981; Xia and Yang, 2019).

At the end of the CFA, the validated measurement model was obtained as a result and an evaluation of the goodness of fit of the measurement model was carried out separately for the samples of mothers = 1 and fathers = 0, with ordinal indicator variables (Kim and Yoon, 2011). Next, the multigroup invariance analysis of the model resulting from the CFA was investigated. The invariance evaluations included: (0) configural invariance that compares the structure of the factors and the location of the items; (1) metric invariance that evaluates the magnitude of the factor loadings and sense of the saturations; (2) scalar invariance that compares the proportions of the thresholds of the Likert-type response scale; and (3) strict invariance that assesses the residuals or unicities between the empirical correlation matrix and that reproduced by the model. To evaluate compliance with the configural invariance, the mean square error of approximation (RMSEA; Xia and Yang, 2019) and the comparative fit indices (CFI) and the Tucker-Lewis Index (TLI; Hu and Bentler, 1999). A good fit was considered when RMSEA ≤ 0.06, CFI and TLI ≥ 0.95 (Hu and Bentler, 1999) and an acceptable fit was RMSEA ≤ 0.08, CFI and TLI ≥ 0.90 (Kline, 2005). The same goodness-of-fit indicators were used for the CFA. From this stage, the calculation of the Chi-Squared value of goodness of fit begins to be estimated in the likelihood ratio tests between two consecutive nested models. Due to the inverse relationship that exists between the degrees of freedom and the Chi-Square value of *p* (Ong and Van Dulmen, 2007), for the test of significance between the invariance models, the Chi-Square difference test was used for nested models with ordinal categorical variables (DIFF-TEST; Svetina et al., 2020), which must present a value of *p* > 0.05. Therefore, from the evaluation of the metric invariance onwards to the precision and incremental adjustment indices, the test of the significance difference of the Chi-Square DIFF-TEST for ordinal variables was added, which allows evaluating compliance with the invariance of each stage of the analysis. When the invariance was not verified in any of the stages, the partial invariance was calculated. To select the parameter to be estimated in free form, the modification index (MI) that presented the greatest reduction of the Chi-Square value of *p* > 3.84 was identified (Bearden et al., 1982). Once the corresponding parameter was released, the procedure was repeated until reaching a DIFF-TEST *χ*^2^ with value of *p* > 0.05 and the adjustment indicators within the acceptance ranges or higher, until the corresponding partial invariance model was fulfilled. The DIFF TEST is a statistical test for ordinal variables that includes a scaling correction factor for sample size equivalent to the corrected Satorra-Bentler test for continuous variables. This result has been supported by simulations showing that the DIFF-TEST maintained a constant value of *p* of 0.05 with large increases in sample size (Asparouhov and Muthen, 2006).

## 3. Results

### 3.1. Exploratory factor analysis

To identify the homogeneity of the items and the number of factors to retain, the exploratory factor analysis of a five-factor model of the CFPQ was carried out with a subsample of 284 participants corresponding to 30% of the total sample of 946 participants. The sample adequacy was KMO = 0.859, determinant >0.0 and a significant Bartlett sphericity test (*p* ≤ 0.001), which showed the relevance of applying the EFA to the correlation matrix of the variables. Horn’s parallel analysis suggested a dimensionality of four factors as a better explanation of the factorial structure of the five initially proposed factors (Williams et al., 2010).

The Environment factor presented a negative cross correlation with the Restriction for weight control factor item: “I often put my child on a diet to control his / her weight,” in addition the Environment factor lost two of its items “Most of the food I keep in the house is healthy” and “A variety of healthy foods are available to my child at each meal served at home” loaded on the Modeling factor. In this way, the Environment factor was made up only of two inverse items:” I keep a lot of snack food (potato chips, Doritos, cheese puffs) in my house” and “I keep a lot of sweets (candy, ice cream, cake, and pastries) in my house.” Therefore, the Environment factor was eliminated, leaving a measurement model with four factors: Monitoring, Child control, Restriction for weight control and Modeling (Table 2).

**Table 2:** Exploratory factorial analysis of comprehensive feeding practices questionnaire (CFPQ).

Items	Monitoring	Control	Restriction	Modeling	Environment
Mon2	0.999	–	–	–	–
Mon1	0.955	–	–	–	–
Mon3	0.933	–	–	–	–
Mon4	0.894	–	–	–	–
CC6	–	0.785	–	–	–
CC10	–	0.604	–	–	–
CC11	–	0.595	–	–	–
CC12	–	0.525	–	–	–
CC5	–	0.501	–	–	–
RW33	–	–	0.878	–	–
RW29	–	–	0.852	–	–
RW27	–	–	0.848	–	–
RW34	–	–	0.795	–	–
RW35	–	–	0.74	–	–
RW41	–	–	0.689	–	–
RW45	–	–	0.59	–	−0.34
RW18	–	–	0.417	–	–
Mod48	–	–	–	0.925	–
Mod44	–	–	–	0.821	–
Mod46	–	–	–	0.82	–
Mod47	–	–	–	0.795	–
Env14	–	–	–	0.498	–
Env37	–	–	–	–	0.755
Env22	–	–	–	0.448	–
Env16	–	–	–	–	0.751

Prefix’ items are labeled as follows: Monitoring (Mon), Child control (CC).

Restriction for weight control (RW), Modeling (Mod), Environment (Env).

### 3.2. Confirmatory factor analysis

To verify the factor structure that resulted from the EFA and to evaluate the reliability and validity of the measurement model of the four retained factors, a CFA was applied in the validation subsample of 662 participants, corresponding to 70% of the total sample of 946. In the CFA, two items with factor loadings < 0.4 of the Child control factor were eliminated: “If this child does not like what is being served, do you make something else?” and “Do you allow this child to leave the table when s/he is full, even if your family is not done eating?.” After this, the measurement model presented an acceptable global fit (RMSEA = 0.073) and good incremental fit indices (CFI = 0.985, TLI = 0.983). The convergent validity was confirmed with significant estimates (*p* ≤ 0.001) of the factor loadings. Although this test is sensible to the sample size, these results align with those from previous research with samples sizes that are smaller (Mais et al., 2015; Warkentin et al., 2016; Piccoli et al., 2017; Saltzman et al., 2018; Arlinghaus et al., 2019; Hidalgo-Mendez et al., 2019; Ángel et al., 2021) and larger (Haszard et al., 2013; Al-Qerem et al., 2017) than the sample size in this study. All four factors met the discriminant validity. Reliability was evaluated with McDonald’s Omega coefficient, presenting good values between 0.74 and 0.97, with most of the asymmetry and kurtosis indices corresponding to non-normal asymmetric distributions (Table 3). Thus, a model with four factors and 19 items was confirmed.

**Table 3:** CFA, standardized loadings and descriptive characteristics by mothers and fathers samples.

		Mothers (*n* = 473)					Fathers (*n* = 473)				
Factors	Items	Loadings	Mean	SD	Skewness	Kurtosis	Loadings	Mean	SD	Skewness	Kurtosis
Monitoring	Mon1	0.943	3.81	1.19	–0.85	–0.18	0.965	3.11	1.33	–0.17	–1.13
	Mon2	0.969	3.84	1.21	–0.92	–0.07	0.971	3.14	1.34	–0.18	–1.14
	Mon3	0.904	3.67	1.22	–0.71	–0.45	0.919	3.07	1.34	–0.10	–1.16
	Mon4	0.896	3.77	1.26	–0.80	–0.44	0.888	3.12	1.36	–0.16	–1.18
Control	CC1	0.779	2.47	1.04	0.43	–0.21	0.883	2.68	1.16	0.37	–0.53
	CC2	0.533	1.23	1.05	0.57	–0.26	0.445	2.29	1.12	0.55	–0.41
	CC3	0.679	2.34	1.03	0.59	–0.05	0.776	2.61	1.15	0.38	–0.53
Restriction for weight	RW18	0.524	3.46	1.43	0.49	1.12	0.543	3.33	1.42	–0.36	–2.00
	RW27	0.821	3.49	1.50	–0.61	–1.08	0.813	3.44	1.42	–0.56	–1.00
	RW29	0.855	3.03	1.46	–0.17	–1.36	0.810	2.88	1.38	–0.08	–1.25
	RW33	0.847	3.07	1.50	–0.20	–1.41	0.827	2.92	1.45	–0.06	–1.36
	RW34	0.830	3.56	1.35	–0.76	–0.60	0.895	3.39	1.38	–0.57	–0.90
	RW35	0.762	3.75	1.38	–0.92	–0.42	0.720	3.62	1.33	–0.76	–0.55
	RW41	0.701	3.01	1.37	–0.14	–1.18	0.721	2.94	1.39	–0.07	–1.24
	RW45	0.526	1.96	1.28	1.01	–0.32	0.609	2.09	1.30	–0.75	–0.79
Modelling	Mod44	0.770	3.88	1.09	–1.02	0.58	0.827	3.55	1.28	–0.64	0.63
	Mod46	0.764	3.67	1.32	–0.73	–0.64	0.874	3.49	1.40	–0.66	–0.84
	Mod47	0.875	4.37	0.94	–1.84	3.45	0.876	4.06	1.09	–1.32	1.26
	Mod48	0.860	4.12	1.08	–1.34	1.37	0.991	3.79	1.24	–0.88	–0.17

### 3.3. Multigroup invariance analysis for the measurement model

In the sequential measurement invariance verification procedure, six models were evaluated to verify the equivalence of the measurement between the two subsamples for father and mothers.

The results of the adjustments of the four-factor model in the sample of mothers presented the following values: *χ*^2^ (*df*) = 454.753 (146), *p* ≤ 0.001, RMSEA = 0.067, CFI = 0.984, TLI = 0.982 and for the sample of fathers: *χ*^2^ (*df*) = 562.286 (146) *p* ≤ 0.001, RMSEA = 0.078, CFI = 0.986, TLI = 0.984. Model 0, or configural, showed a good fit (CFI = 0.952; TLI = 0.953; RMSEA = 0.059), confirming the instrument’s configural invariance between samples of fathers and mothers. This result was used as a basis to evaluate the equality of the factor loadings corresponding to the invariance of Model 1 (metric invariance). The DIFF-TEST test of difference between model 0 and 1 (*χ*^2^ (15) = 15.918, *p* = 0.388) verified the metric invariance. The DIFF-TEST of difference *χ*^2^ between Model 1 (metric invariance) and Model 2 (scalar invariance; *χ*^2^ (50) = 60.004, *p* = 0.039), did not allow to confirm the invariance of model 2. The evaluation of MI led to release the estimation of the parameter of the second threshold of item 8 of the factor Restriction for weight control “I often put my child on a diet to control his/her weight?.” Mothers disagreed with this item more frequently than fathers. The estimation of the invariance of the partial model 2 partial 1 (Model 2P1; Table 4) with the free parameter allowed to confirm the scalar invariance, through the *χ*^2^ test of difference between the scalar model and the partial scalar model 1 (1P; *χ*^2^ (49) = 58.875, *p* = 0.158). Finally, strict invariance was not confirmed with *p* = 0.039 and the highest MI was identified, releasing the residual of item 2 of the Modeling factor “I try to eat healthy foods in front of my child, even if they are not my favorite?,” thus, achieving the strict invariance of model 3 partial 1 (Model 3P1; Table 4) with good incremental adjustment indicators (CFI = 0.963; TLI = 0.966; RMSEA = 0.046).

**Table 4:** Multigroup measurement model invariance analysis with ordinal categorical indicators variables.

Model of invariance	*χ* ^2^	*df*	D DIFF-TEST	D *df*	p DIFF-TEST	RMSEA	CFI	TLI
Model 0: Configural invariance (all lodings and thresholds and residuals are freely estimated)	777.986	292	–	–	–	0.059	0.952	0.953
Model 1: Metric invariance (loadings are fixed across groups, thresholds and residuals are freely estimated)	741.996	307	15.918	15	0.388	0.055	0.957	0.952
Model 2: Scalar invariance (loadings and thresholds are fixed across groups and residuals are freely estimated)	767.538	357	69.004	50	0.039	0.049	0.959	0.961
Model 2P1: Scalar partially invariance 1 (loadings and thresholds are fixed across groups, with threshold 2 of items 8 is freely estimated and residuals are freely estimated)	756.078	356	58.875	49	0.158	0.049	0.960	0.962
Model 3: Strict invariance (loadings, thresholds and residual variance are fixed across groups, with threshold 2 of item 8 is freely estimated)	756.204	375	31.049	19	0.039	0.046	0.962	0.965
Model 3P1: Strict partially invariance 1 (loadings, thresholds and residual variance are fixed across groups, with threshold 2 of item 8 and residual variance of item 2 is freely estimated)	748.766	374	24.727	18	0.133	0.046	0.963	0.966

*χ*^2^ = Chi-square.

*df* = degrees of freedom.

D DIFF-TEST = Delta of DIFF-TEST between goodness of fit test a model and previous model.

D *df* = Delta of DIFF-TEST degrees of freedom.

p DIFF-TEST = Statistical significance of Delta of DIFF-TEST.

RMSEA = root mean square error of approximations.

CFI = comparative fit index.

TLI = Tucker-Lew.

## 4. Discussion and conclusions

The original version of the CFPQ Musher-Eizenman and Holub (2007) Was developed in English and with Caucasian families, which has made its cultural and idiomatic validation necessary in other contexts (Arlinghaus et al., 2019). Research with this instrument has been conducted mainly in English-speaking countries in North America and Europe (Musher-Eizenman and Holub, 2007; Melbye et al., 2011, 2012; Haszard et al., 2013; Saltzman et al., 2018) and to a lesser extent in South America (Mais et al., 2015; Warkentin et al., 2016; Piccoli et al., 2017) and Asia (Al-Qerem et al., 2017). Although recent research evaluated the psychometric properties of the instrument in samples of mothers of Hispanic origin (Arlinghaus et al., 2019) or adapted it to Spanish to be used with Mexican mothers (Ángel et al., 2021), none of these studies considered both parents, or considered parents with teenage children. Therefore, this is the first investigation to adapt and evaluate the psychometric properties of the version of the CFPQ adapted by Melbye et al. (2011) to be answered by parents with adolescent children in a Spanish-speaking Latin American country. It is also noteworthy that this research was carried out with Likert-type ordinal response variables.

In this research, five factors and 25 items were selected from the CFPQ adapted by Melbye et al. (2011), based on the stability of the factors in previous research (Haszard et al., 2013; Mais et al., 2015; Saltzman et al., 2018; Arlinghaus et al., 2019) and evidence that accounts for the use of selected FPPs in parents of adolescents (Loth et al., 2016; Schnettler et al., 2018b; Gunther et al., 2019; Garrido-Fernández et al., 2020; Haycraft, 2020; Lister et al., 2020; Costarelli et al., 2021; Jaeger et al., 2021; Schnettler et al., 2021). The reduction in the number of factors and items of the CFPQ instrument obtained through the EFA and CFA in this investigation is consistent with other investigations that report the lack of adjustment of items and factors of the original instrument. In this research, the environment factor was eliminated due to a lack of internal consistency, high homogeneity or similarity with the meaning of the items of the Monitoring factor and, a significantly high correlation with the Modeling factor. For a similar reason, the Environment factor has been eliminated or integrated into other factors in previous studies with adolescents and young children (Melbye et al., 2011; Haszard et al., 2013; Mais et al., 2015; Warkentin et al., 2016; Piccoli et al., 2017; Saltzman et al., 2018; Arlinghaus et al., 2019). In addition, it should be noted that in our sample only the items “I keep a lot of snack food (potato chips, Doritos, cheese puffs) in my house” and “I keep a lot of sweets (candy, ice cream, cake, and pastries) in my house” remained in the Environment factor, which did not sufficiently capture the meaning of the Environment factor, i.e., “assessment of how parents make (un)healthy foods available in the home.” These two items only capture the availability of unhealthy foods at home and not the availability of healthy foods. Therefore, future research should revise the items of the Environment factor, proposing items that correctly conceptualize this FPP from a theoretical point of view.

The CFA applied to the final four-factor model resulted in the elimination of two items of the child control factor that presented loadings < 0.40 (“Do you allow this child to leave the table when s / he is full, even if your family is not done eating?” and “If this child does not like what is being served, do you make something else?). This reduction of items is consistent with previous research in different countries and languages (Melbye et al., 2011; Haszard et al., 2013; Arlinghaus et al., 2019). Therefore, in the present investigation the following factors were retained: Monitoring with 4 items, Child control with 3 items, Restriction for weight control with 8 items and Modeling with 4 items. The reduction in the number of factors achieved in this research contributes to parsimony (Haszard et al., 2013), ease of interpretation and understanding of the FPP, while the analysis with a large sample contributes to the knowledge with greater coverage, precision and projection of the factors retained from the CFPQ. The evaluation of the psychometric properties of the measurement model resulting from four factors and 19 items of the CFPQ presented convergent and discriminant validity and showed medium and high levels of reliability and goodness of fit. Therefore, the version adapted to Spanish of four factors of the CFPQ to be answered by parents of adolescents is a valid and reliable instrument for assessing monitoring, modeling, restriction for weight control and child control in Chilean parents.

Barragán et al. (2018) emphasize the need for more research related to eating habits with a gender perspective, since the studies have not really delved into the differences between men and women. On the other hand, previous research indicates that there are differences in how fathers and mothers apply FPP (Frankel and Kuno, 2019; Rahill et al., 2020), and consider it inappropriate to extrapolate the results obtained in mothers to fathers (Frankel and Kuno, 2019). This is the first study that provides evidence regarding the measurement invariance of the version of the CFPQ adapted by Melbye et al. (2011), according to the gender of the parents. The results of the invariance analysis evidenced compliance with the configural and metric invariance, which means that mothers and fathers have a common perception of the structure of the 19 items in the four retained factors. The fulfillment of the metric invariance allowed to verify that the factor loadings are invariant between mothers and fathers.

The scalar invariance evidenced that the threshold of the second rank of Likert-type ordinal response (2 = in part disagreement) of the item “I often put my child on a diet to control his/her weight?” of the Restriction for weight control factor is not invariant, this threshold presenting a higher frequency of responses in the sample of mothers than in fathers. This result would indicate that mothers are less in agreement with putting their children on a diet than fathers. This finding is in line with previous research that reports that Latino mothers underestimate the weight of their children (Hidalgo-Mendez et al., 2019). In this regard, some research suggests that in Latin America thinness can be interpreted as a sign of disease. Thus, an overweight child would show good parents who have the ability to keep him “well fed” (Arlinghaus et al., 2019). However, it is also possible that this different response pattern is related to the tendency of fathers to apply FPPs more coercively than mothers (Rahill et al., 2020).

In relation to the equivalence of the residuals, item 2 of the Modeling factor “I try to eat healthy foods in front of my child, even if they are not my favorite?” was identified as non-invariant. In this research, it was evidenced that fathers agreed less than mothers with this item. In this regard, there is evidence that indicates the existence of differences between what parents do and what they report on eating habits (Schnettler et al., 2018c). Piccoli et al. (2017) state that the strategies to show healthier behaviors to their children are applied less by fathers than by mothers.

Among the limitations of this research, it is found that it was developed in a single Latin American Spanish-speaking country, using a cross-sectional design and a non-probabilistic sample. Therefore, future research should consider representative samples of the population, cross-cultural studies and use a longitudinal design. The latter would allow evaluating the change in FPP over time (Jansen et al., 2016; Saltzman et al., 2018) allowing the measurement of the effects of how mothers and fathers contribute to the eating habits of their children (Frankel and Kuno, 2019). Likewise, the results of scalar and strict partial invariance suggest studying the evolution of these properties using a longitudinal invariance analysis, which allows including the correlation of the residuals over time, correcting the strict partial invariance determined by the greater dispersion of the behaviors of the Modeling factor. In the case of scalar partial invariance, it is proposed to perform cluster analysis over time, through an analysis of transition profiles. This would make it possible to identify types of mothers and fathers with different frequencies in the Likert-type response range of the item “I often put my child on a diet to control his/her weight” of the factor Restriction for weight control factor.

Notwithstanding the foregoing, the results of this research confirmed the adaptation, validation of the psychometric properties and the measurement invariance of the four-factor model of the CFPQ instrument at the level of the configuration of the factor structure and factor loadings between samples of mothers and fathers with adolescent children in a Spanish-speaking context. This constitutes a relevant element to measure factor scores in the four-factor model that are associated with the promotion of eating habits in adolescent children in the family context, making it possible to establish associations with characteristics of parents and children, such as age, gender, and socioeconomic level. This is relevant to carry out future interventions that improve the quality of the diet of families with adolescent children, especially in families with double income because workers’ high job demands have been associated with lower diet quality for the worker and their families, as personal resources such as time and energy are invested in workplace responsibilities in detriment of food preparation and consumption (Djupegot et al., 2017; Takeda et al., 2018; Garrido-Fernández et al., 2020).

## Data availability statement

The original contributions presented in the study are included in the article/Supplementary material, further inquiries can be directed to the corresponding author.

## Author contributions

CD wrote the first draft of the manuscript. BSch designed the study and procedures, coordinated study and data collection. CD and HM performed the data analyses. BSch and LO conducted the research and revised manuscript drafts. KG and HM provided a critical analysis of the study throughout all stages. All authors contributed to the article and approved the submitted version.

## Funding

This study was funded by ANID, Fondecyt Project no. 1190017.

## Conflict of interest

The authors declare that the research was conducted in the absence of any commercial or financial relationships that could be construed as a potential conflict of interest.

## Publisher’s note

All claims expressed in this article are solely those of the authors and do not necessarily represent those of their affiliated organizations, or those of the publisher, the editors and the reviewers. Any product that may be evaluated in this article, or claim that may be made by its manufacturer, is not guaranteed or endorsed by the publisher.
