# Supplementary material for: Measurement invariance of the comprehensive feeding practices questionnaire in dual-earner parents with adolescent children in Chile
Source: Front Psychol. 2023 Jan 4;13:1031391. doi: 10.3389/fpsyg.2022.1031391 (PMC9846088; doi:10.3389/fpsyg.2022.1031391)
Supplement: Supplementary file 1 [file Table_2.DOCX]

**Supplementary Material.**

**Adaptation of CFPQ from English to Spanish. Items’ suffixes are labeled according to Melbye et al. (13).**

| **Factors** | **Items** |
| --- | --- |
| **Monitoring** | How much do you keep track of the sweets (candy, ice cream, cake, pastries) that you child eats? (Mon1) |
|  | ¿Con qué frecuencia revisa la cantidad de dulces (helados, pasteles, chocolates, caramelos, pies, queques) que come su hijo/a? |
|  | How much do you keep track of the snack food (potato chips, Doritos, cheese puffs) that your child eats? (Mon2) |
|  | ¿Con que frecuencia revisa la cantidad de snacks (papas fritas, ramitas, doritos) que come su hijo/a? |
|  | How much do you keep track of the high-fat foods that your child eats? (Mon3) |
|  | ¿Con qué frecuencia revisa la cantidad de alimentos altos en grasas que come su hijo/a? |
|  | How much do you keep track of the sugary drinks this child drinks? (Mon4) |
|  | ¿Con qué frecuencia revisa la cantidad de bebidas azucaradas (bebidas, jugos) que su hijo/a toma? |
| **Child Control** | Do you let your child eat whatever s/he wants? (CC5) |
|  | ¿Deja que su hijo/a coma lo que quiera? |
|  | At dinner, do you let this child choose the foods s/ he wants from what is served? (CC6) |
|  | En la cena, ¿le permite a su hijo/a elegir los alimentos que quiere comer? |
|  | If this child does not like what is being served, do you make something else? (CC10) |
|  | ¿Si a su hijo/a no le gusta lo que hay para comer, ¿prepara algo más? |
|  | Do you allow this child to eat snacks whenever s/he wants? (CC11) |
|  | ¿Le permite a su hijo/a comer snacks cuando lo desee? |
|  | Do you allow this child to leave the table when s/ he is full, even if your family is not done eating? (CC12) |
|  | ¿Le permite a su hijo/a dejar la mesa cuando está satisfecho, incluso si el resto de la familia no ha terminado de comer? |
| **Restriction for weight control** | I have to be sure that my child does not eat too many high-fat foods (RW18) |
|  | Tiene que asegurarse de que su hijo/a no coma demasiados alimentos altos en grasa |
|  | I encourage my child to eat less so s/he won’t get fat (RW27) |
|  | Anima a su hijo/a a comer menos para que no suba de peso |
|  | I give my child small helpings at meals to control his/her weight (RW29) |
|  | Le da a su hijo/a pequeñas porciones de comidas para controlar su peso |
|  | If my child eats more than usual at one meal, I try to restrict his/her eating at the next meal (RW33) |
|  | Si su hijo/a come más de lo normal en una comida, trata de restringir la cantidad que come en la siguiente comida |
|  | I restrict the food my child eats that might make him/her fat (RW34) |
|  | Restringe la comida que podría hacer engordar a sus hijos/as |
|  | There are certain foods my child shouldn’t eat because they will make him/her fat (RW35) |
|  | Hay ciertos alimentos que su hijo/a no debería comer porque lo harán engordar |
|  | I don’t allow my child to eat between meals because I don’t want him/her to get fat (RW41) |
|  | No permite que su hijo/a coma entre comidas porque no quiere que engorde |
|  | I often put my child on a diet to control his/her weight (RW45) |
|  | A menudo pone a su hijo/a a dieta para controlar su peso |
| **Modeling** | I model healthy eating for my child by eating healthy myself (Mod44) |
|  | Come alimentos saludables para dar el ejemplo de una alimentación saludable a mis hijos |
|  | I try to eat healthy foods in front of my child, even if they are not my favorite (Mod46) |
|  | Trata de comer alimentos saludables frente a su hijo/a, incluso si no son sus favoritos |
|  | I try to show enthusiasm about eating healthy foods (Mod47) |
|  | Trata de entusiasmar a sus hijos respecto de comer alimentos saludables |
|  | I show my child how much I enjoy eating healthy foods (Mod48) |
|  | Le muestra a su hijo/a como disfruta comer comida saludable |
| **Environment** | Most of the food I keep in the house is healthy (Env14) |
|  | La mayoría de la comida que tiene en la casa es saludable |
|  | I keep a lot of snack food (potato chips, Doritos, cheese puffs) in my house R (Env16) |
|  | Tiene muchos snacks (papas fritas, ramitas, doritos, palomitas de maíz saladas) en su casa |
|  | A variety of healthy foods are available to my child at each meal served at home (Env22) |
|  | Hay una variedad de alimentos saludables disponibles para su hijo/a en cada comida que se sirve en casa |
|  | I keep a lot of sweets (candy, ice cream, cake, pastries) in my house R (Env37) |
|  | Tiene muchos dulces (helados, postres, golosinas, queque, caramelos) en su casa |

Labels: Monitoring (Mon), Child control (CC), Restriction for weight control (RW), Modeling (Mod), Environment (Env).

Reversed coded = R
